# Supplementary figures and images for: Distinct lipid transport proteins are regulated by innate immune stimuli
Source: Discov Immunol. 2026 Jul 2;5(1):kyag014. doi: 10.1093/discim/kyag014 (PMC13371113; doi:10.1093/discim/kyag014)

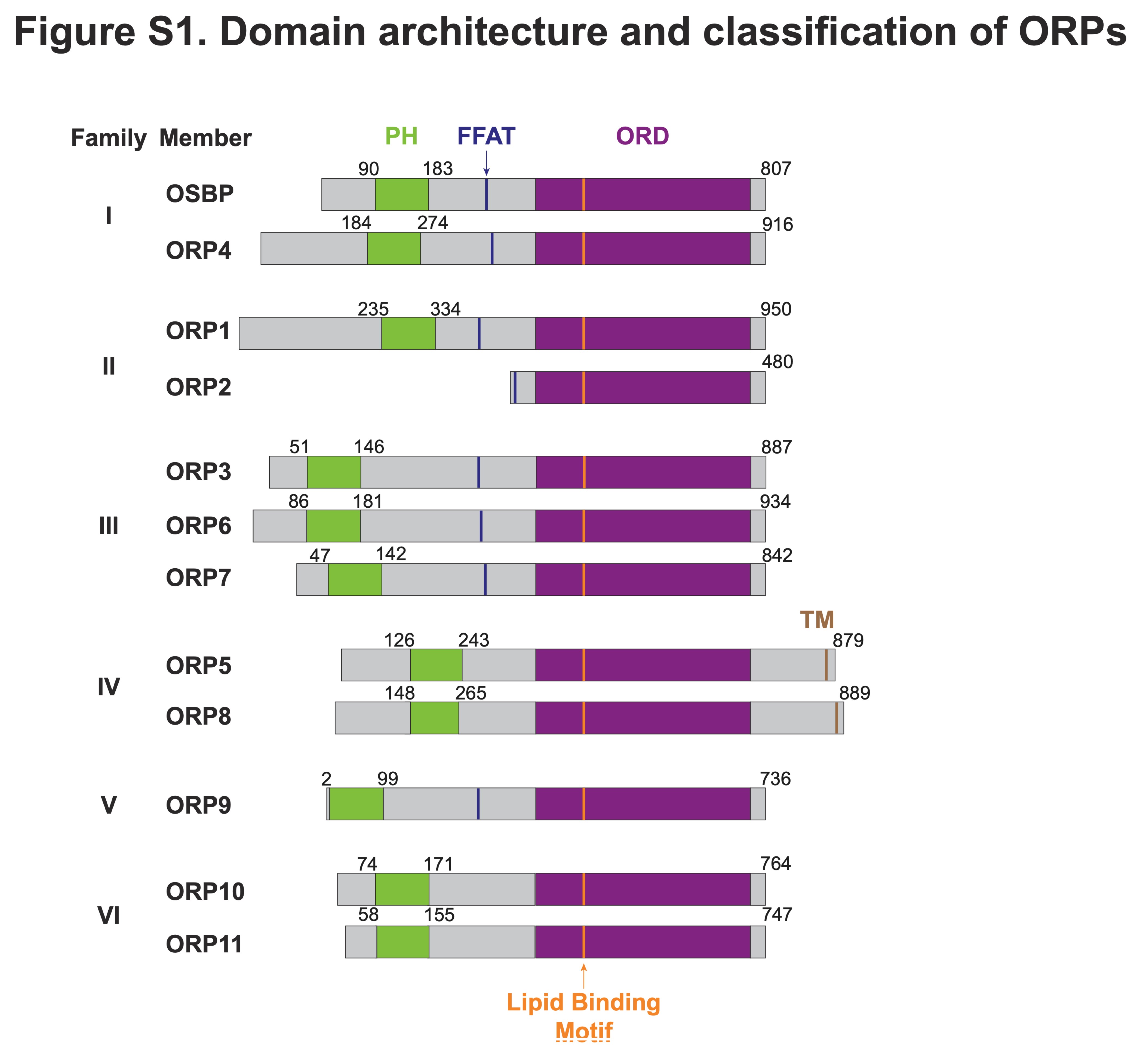

Supplement: kyag014_Supplementary_Data [file kyag014_supplementary_data.jpeg]
